# With or without U(K): A pre-Brexit network analysis of the EU ETS

**DOI:** 10.1371/journal.pone.0221587

**Published:** 2019-09-09

**Authors:** Simone Borghesi, Andrea Flori

**Affiliations:** 1 FSR Climate, European University Institute, Florence, Italy; 2 Department of Political and International Sciences, University of Siena, Siena, Italy; 3 Department of Management, Economics and Industrial Engineering, Polytechnic University of Milan, Milan, Italy; Universita Cattolica del Sacro Cuore, ITALY

## Abstract

The European Emission Trading System (EU ETS) is commonly regarded as the key pillar of the European climate policy and as the main unifying tool to create a unique carbon price all over Europe. The UK has always played a crucial role in the EU ETS, being one of the most active national registry and a crucial hub for the exchange of allowances in the market. Brexit, therefore, could deeply modify the number and directions of such exchanges as well as the centrality of the other countries in this system. To investigate these issues, the present paper exploits network analysis tools to compare the structure of the EU ETS market in its first two phases with and without the UK, investigating a few different scenarios that might emerge from a possible reallocation of the transactions that have involved UK partners. We find that without the UK the EU ETS network would become in general much more homogeneous, though results may change focusing on the type of accounts involved in the transactions.

## Introduction

The implications of Brexit are today the object of a heated debate and have gained much attention in the public opinion, both in the UK and in the rest of Europe. Among the many different consequences that Brexit could have, an important aspect concerns its impact on the EU climate and energy policies and, in particular, on the European Emission Trading Scheme (henceforth EU ETS) that represents the cornerstone of the EU policy to fight climate change. The EU ETS was in fact deployed in January 2005 as the first transboundary cap-and-trade scheme and nowadays covers more than 11,000 installations from several emission-intensive sectors and across 31 States (the 28 EU Member States plus Norway, Iceland and Liechtenstein). Overall, these sectors account for about 50% of the total European CO2 emissions and 45% of all GHG emissions [1]. The EU ETS was originally divided in three phases: Phase I from 2005 to 2007, Phase II from 2008 to 2012, and Phase III from 2013 to 2020, while a new Directive [2] has been recently adopted to reform the EU ETS for Phase IV (2021-2030). The EU ETS represents the largest ETS in the world and has stimulated the adoption of similar ETS in several other regions [3, 4] (e.g., Alberta and Quebec in Canada, China, Japan, Kazakhstan, South Korea, California and the Eastern part of the US).

The possible effects that Brexit could have for the EU ETS have been mainly ignored so far. Nevertheless, in our opinion, the Brexit effect on the structure and effectiveness of the EU ETS deserves greater attention being of crucial importance for the effectiveness of this instrument and for the future design of both the EU and UK climate policies. At the moment of writing the outcome of the UK-EU negotiations on the UK exit from the EU ETS appears still rather uncertain. In November 2017, UK and EU agreed that UK emitters will have to surrender carbon units before the scheduled Brexit date. In March 2018, negotiators reached a deal on a transition period to the end of 2020, during which the UK will no longer participate in EU decision-making processes but will still be subject to the single market rules [5].

Some timely studies have started to examine how Brexit could affect the EU-UK relationships in terms of their climate and energy policies. For instance, changes in the UK climate policies following the vote to leave have been found to be likely to have small global economic consequences given the limited amount of UK emissions [6], but still generating a surplus of allowances in the short-term, since UK companies would want to sell their allowances that are no longer needed, and a tightening of the system in the long term [7]. In addition, studies focusing on the neighbouring states that have physical energy interconnections with the UK indicate that Brexit would have limited impact on gas and electricity prices both in UK and EU [8]. Assuming the extension of the EU ETS to non-ETS sectors in the future, numerical simulations find that a hard Brexit could have a negative effect on the UK’s climate policy costs and a positive one on the remaining EU member states [9]. As discussed in [10], the impact of Brexit on the remaining 27 member states would be limited if the EU accepts a weaker emissions cap. On the contrary, such impact is likely to be much larger for the UK in terms of increased compliance costs with its climate policy targets (estimated to range between 0.2 and 0.4 percent of its GDP), transition costs to replace the EU ETS on short notice, possible business loss as the carbon trade leaves London (that played a pivotal role as a relevant hub in the system so far), and distortions at the border due to differences between UK and EU GHG regulations.

No one has investigated so far the potential effects that Brexit could have on the structure of the EU ETS itself. The UK, in fact, plays a crucial role within the EU ETS, being one of the most active national registries with about 1,000 accounts actively involved in the exchange of allowances in the market, facilitated also by the presence of a key devoted platform for trading permits (namely, the Intercontinental Exchange—ICE). Brexit, therefore, could deeply modify the number and directions of such transactions as well as the centrality of the other registries operating in the system.

To investigate these issues, the present paper examines the structure of the EU ETS market with and without the UK, using network analysis instruments. Network theory can potentially be used to study many environmental topics [11], such as the structure of common property resources in the presence of multiple sources and users [12], how social interactions affect the adoption of eco-innovation [13], the stability of International Environmental Agreements when pollution has both global and local effects [14], how network structure influences resource exploitation [15] or global commodity trade [16] or how climate variability affects food resource availability [17]. Building upon [18], who analyze the network dynamics of the EU ETS, and [19], who use network theory to describe the structure of the EU ETS at national registry-level, in this paper we will exploit network measures to investigate the impact of Brexit on the EU ETS structure proposing a few different scenarios that might emerge from a possible reallocation of the transactions that are currently involving UK partners. Our findings indicate that, without the UK, the EU ETS would resemble a much more homogeneous network in which a small club of national registries would probably replace the leading role of UK, at least with respect to operations performed by pure trading accounts.

## Materials and methods

### Data: EU ETS transactions and account types

Data are retrieved from the *European Union Transaction Log—EUTL*, the European infrastructure containing all available information on the transactions under the EU ETS (http://ec.europa.eu/environment/ets/transaction.do). Transactions in the EU ETS can be categorized along at least two main dimensions: i) the type of the counterparts involved in the trade, and ii) the transaction type. As to the first dimension, participants in the EU ETS can be either compliance liable entities that refer to installations responsible for greenhouse gases emissions (named, “Operator Holding Accounts”—OHAs) or voluntary accounts that operate mainly for trading purposes (named, “Person Holding Accounts”—PHAs); in addition, a bundle of players refers to governmental accounts through which allowances are managed for compliance purposes. As to the second dimension, transactions may be distinguished either in terms of internal vs. external exchanges (i.e., within the same national registry or across different registries) or for the reason underlying the transaction (e.g., trade, issuance, allocation, surrendering, cancellation, correction, etc.).

In this analysis we refer to the period from January 2005 to December 2012 in order to completely include two compliance phases, namely both Phase I and Phase II of the program. In this interval, EU ETS transactions amounted to 656, 735 operations corresponding to 155, 823, 895, 749 transferred units (see Table 1). Total external transactions were 155, 555 (equivalent to about 23.68 per cent of the overall transactions) involving 14, 922, 967, 382 units being transferred. Total internal transactions were 498, 209 (75.86 per cent of all transactions) corresponding to 91, 530, 558, 100 units being transferred. Transactions involving OHAs and PHAs represented about 43 per cent of the transferred amount. In that period, UK transferred 26, 617, 737, 094 units and received 27, 492, 932, 700 allowances. Hence, it was responsible for more than 17 per cent of the traded units as either transferring or acquiring registry. These figures confirm the relevant role of UK as a very active registry within the EU ETS.

**Table 1:** Descriptive statistics: EU ETS. First column shows the description of each transaction type. The second column indicates the codes corresponding to the transaction type. The third column reports the number of transactions for each type. The fourth column shows the amount of transferred allowances. Source: authors’ own elaborations based on the EUTL transactions data set for the first two Phases.

Explanation	Transaction Type	# of Transactions	# of Units
***Issuance***	***code 1***	***321***	***34,848,385,716***
***Conversion***	***code 2***	***732***	***71,145,927***
***External Transfer***	***code 3***	***155,555***	***14,922,967,382***
*External Transfer*	3-0	139,966	13,887,754,931
*External Transfer—Allowance surrender*	3-2	117	25,424,725
*External Transfer (2005-2007)*	3-21	15,472	1,009,787,726
***Cancellation***	***code 4***	***1,679***	***6,031,053,181***
***Retirement***	***code 5***	***239***	***8,419,785,443***
***Internal Transfer***	***code 10***	***498,209***	***91,530,558,100***
*Internal Transfer*	10-0	325,368	42,560,619,951
*Internal Transfer—Allowance Cancellation (2005-2007)*	10-1	3,286	76,877,305
*Internal Transfer—Allowance Surrender*	10-2	85,837	14,038,141,353
*Internal Transfer—Issuance/Internal Transfer Art 63a*	10-24	4	1,011,231
*Internal Transfer—Conversion of Art. 63a Allowances*	10-26	20	508,510
*Internal Transfer—Allocation of Aviation Allowances*	10-35	342	146,831,820
*Internal Transfer—Allocation of General Allowances*	10-36	291	32,173,776
*Internal Transfer—Auction Delivery*	10-37	24	92,201,500
*Internal Transfer—Cancellation and Replacement*	10-41	20	272,312,173
*Internal Transfer—Allowance Issue (2008-2012 onwards)*	10-52	273	10,988,834,103
*Internal Transfer—Allowance Allocation*	10-53	82,376	16,261,299,127
*Internal Transfer—Correction to Allowances*	10-55	8	4,114,611
*Internal Transfer—Surrendered Allowance Conversion*	10-61	164	6,851,333,407
*Internal Transfer—Deletion of Allowances*	10-90	14	174,319,601
*Internal Transfer—Reversal of Allowance Surrender*	10-92	130	19,493,569
*Internal Transfer—Correction*	10-93	51	1,316,081
*Internal Transfer—Reversal of Allowance Cancellation*	10-104	1	9,169,982
**Total**		***656,735***	***155,823,895,749***

In that period, the EU ETS was composed by the following national registries, each represented as a node in the network: AT (Austria), BE (Belgium), BG (Bulgaria), CH (Switzerland), CY (Cyprus), CZ (Czech Republic), DE (Germany), DK (Denmark), EE (Estonia), ES (Spain), FI (Finland), FR (France), UK (United Kingdom), GR (Greece), HU (Hungary), IE (Ireland), IS (Iceland), IT (Italy), LI (Liechtenstein), LT (Lithuania), LU (Luxembourg), LV (Latvia), MT (Malta), NL (Netherlands), NO (Norway), PL (Poland), PT (Portugal), RO (Romania), SE (Sweden), SI (Slovenia), SK (Slovakia), UA (Ukraine). We represent with a separate node the allowances managed by the EC (European Commission), and we create the residual player *RoW* to include: (i) non-EU countries having a marginal role in the system, such as AU (Australia), JP (Japan), NZ (New Zealand), RU (Russian Federation), and (ii) allowances related to CDM (Clean Development Mechanism), the Kyoto Protocol mechanism providing allowances that may be traded in an ETS in exchange for emission reductions projects implemented in developing countries.

### Network representation

Network theory techniques have been applied to study the features of a wide variety of systems (see e.g., [20] and [21]). Economic systems can be represented as a graph or network *G* = (*V*, *E*), where *V* are the nodes representing the agents operating in the system and *E* stands for the set of relationships connecting pairs of nodes. In our framework, each node *i* in *V* refers to a national registry, while the directed link (*i*, *j*) in *E* is weighted according to the number of exchanged allowances from the transferring national registry *i* to the acquiring national registry *j*. The structure of the network is thus summarized by the adjacency matrix *W*, where *W*_*ij*_ = 0 if there is not a link from *i* to *j*, while is *W*_*ij*_ = *w*_*ij*_ if such link exists and corresponds to the amount of allowances *w*_*ij*_ transferred from *i* to *j*.

To capture differences between the two Phases, we consider network representations for the intervals 2005-07 (Phase I) and 2008-12 (Phase II), separately. We focus on either “pure trade” transactions only (i.e., external transactions, codes 3-0 and 3-21, and internal transactions, code 10-0; hereinafter, the *Trade* specification) or the entire list of transaction types which includes also, for instance, the issuance, allocation and surrendering of the allowances (hereinafter, the *All* specification). In addition, we split data according to the two main account types, thus focusing only on PHAs or OHAs.

To characterize the EU ETS we have applied topological measures of the nodes and network properties for the whole graph (for details on network centrality measures see [21–23], among others). Both the degree and the strength scores (and similarly their in-out variants) provide a preliminary representation of the structure of the network based on the amount of links, and possibly their weights, among connected nodes. For instance, a node with a high in-degree refers to a registry which is able to attract transactions from many other registries of the system, while a node with high out-strength and low in- strength stands for a registry more active in transferring allowances than in acquiring them. Betweenness, closeness and eigenvector are also applied to enrich the characterization of the nodes by means of the whole configuration of the network and, in particular, of the neighborhood of each node. A node with a high value of betweenness suggests that it plays a role similar to an intermediary between many other nodes in the network, while a high value for closeness indicates that the node is likely to trade with other nodes directly. Instead, the eigenvector centrality poses importance not only in the amount of incoming links (as approximated for instance by the in-strength of the node), but it also considers how this node is connected to its neighbourhoods. As regards the network as a whole, we compute the assortativity coefficient to analyze the tendency to form connections among “similar” nodes, while centralization measures are introduced to describe the extent to which the cohesion of the graph is set around specific points. For instance, with respect to the degree distribution, the level of centralization may vary from low values corresponding to an almost complete graph to high values achieved for a star-like configuration. Finally, further topological diagnostic is provided by the diameter, the reciprocity and the transitivity. The first indicates a simple upper bound in the connectivity of the graph, the second shows the level of symmetry in links formation, while the third provides a proxy for the emergence of local clusters in the network.

In the EU ETS, for instance, not liable entities (i.e., PHAs) could opt to open accounts in certain registries according to the presence of favourable account set up requirements, fiscal advantages or the establishment of dedicated exchange platforms. Overall, these aspects can affect how national registries are connected between each other. More generally, since these conditions could have changed over time, they may have contributed to move the EU ETS from a centralized system with a few very active nodes, which were initially facilitated by infrastructure advantages, to a more uniform system.

### Scenarios: With or without UK

We propose the following competing reassignment rules to study the removal of the UK from the EU ETS:

- *No reassignment*: we simply remove all the links in which at least one counterpart refers to UK, but we do not reassign the corresponding amount of transferred allowances to the remaining nodes/registries;
- *Proportional reassignment*: we reassign links with UK as one of the counterpart to the other national registries proportionally to the UK neighborhood. Basically, UK has a set of registries from which it imports allowances (namely, its in-neighborhood) and another set to which it exports them (namely, its out-neighborhood). We allocate those links exiting from UK to registries in its in-neighborhood proportionally to their respective weight in the in-strength of UK, while we assign those links entering to UK to registries in its out-neighborhood proportionally to their respective weight in the out-strength of UK. In formula, given the in-strength of UK as $s_{uk}^{In}=\sum_{j=1}^{N}w_{j,uk}$ and the link from UK to a certain registry *x* belonging to its out-neighborhood (namely, *w*(*uk*, *x*)), then the latter is assigned proportionally to each *j* registry in the in-neighborhood of UK as follows: $\hat{w}\left(j,x\right)=w\left(j,x\right)+w\left(uk,x\right)\times \frac{w_{j,uk}}{s_{uk}^{In}}$, where the first term on the rhs refers to the true link between *j* and *x* and the second term indicates the additional flow related to the proportional reassignment of *w*(*uk*, *x*). Similarly, for the in-flows into UK it will be: $\hat{w}\left(k,i\right)=w\left(k,i\right)+w\left(k,uk\right)\times \frac{w_{uk,i}}{s_{uk}^{Out}}$ (the notation is self-explanatory).
- *Random reassignment*: the reassignment of links with UK as one of the counterpart is performed randomly. This is done by generating 1000 simulated realizations, where transferred allowances referred to UK are reassigned to each combination of the remaining registries according to a weight that is drawn from a uniform distribution.

**Fig 1:**
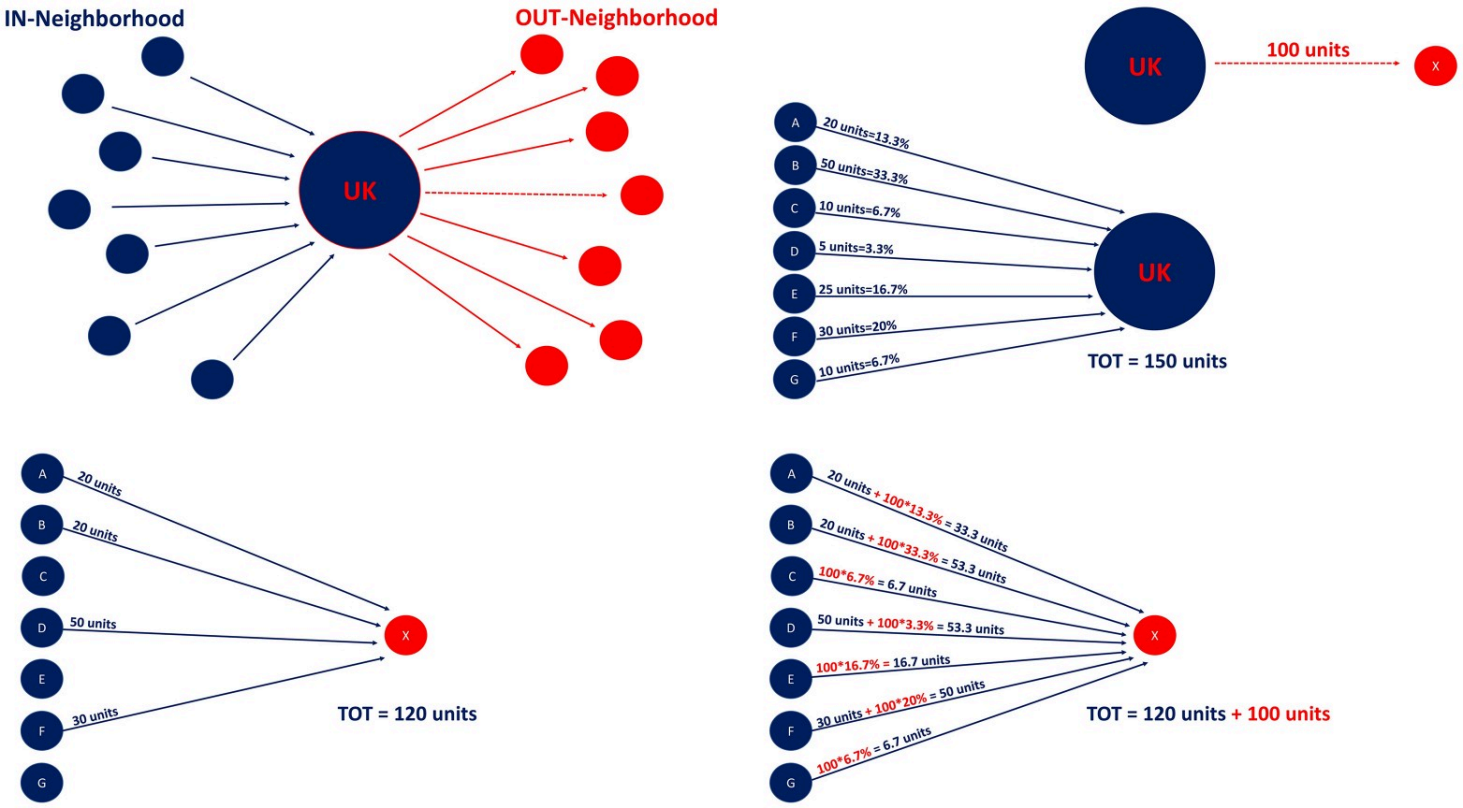
Example: Proportional reassignment. Plot on top-left shows the neighborhood of UK: in blue those registries that transfer units to UK, in red those registries that acquire units from UK. Plot on the top-right isolates in red an outflow from UK to registry *x* (100 units), while in blue indicates the inflows of UK (a total of 150 units from registries A-to-G). Plots on the bottom show the mechanism behind the proportional reassignment of a link exiting from UK. Bottom-left figure reports effective links from registries in the in-neighborhood of UK to registry *x*; bottom-right figure explains that final links from blue nodes to the red one are the sum of the original links plus the proportional assignment of 100 units based on the weight of blue nodes in the inflows connecting them to UK.

For both the *Proportional* and *Random* scenarios we thus analyze a reassignment which considers only transactions with UK as one of the counterpart, while those transactions involving UK as both transferring and acquiring counterparts are discarded (namely, in network jargon we remove the UK self-loop). The latter, in fact, refer to domestic transactions performed by UK accounts, which are therefore less likely to be alternatively operated by other accounts potentially located in other national registries. Fig 1 shows a representative example of the mechanism behind the proportional reassignment, which is considered as the reference scenario in the study.

## Results

As shown in Table 2, the original system (specification *All*) is very dense, transactions between two registries usually go in both directions, and the likelihood these nodes are part of triangles is pretty high. Hence, the EU ETS seems a very connected network and its nodes are likely to trade with many counterparts as both acquiring and transferring peers. Results are very similar if we circumscribe the analysis to the specification *Trade*. Interestingly, we also notice that despite the enlargement of the program to additional national registries (compare, e.g., #N and the diameter), Phase II coincides in general with a more connected network than the one emerging in Phase I. Finally, configurations arising from subsetting the system with only PHAs or only OHAs as both counterparts clearly highlight that the former are more connected than the latter, thus suggesting that not liable entities (i.e., PHAs) are more prone to trade across national registries. This may be due to the fact that PHAs mostly include brokerage firms and financial intermediaries [24], which can actually facilitate transactions across different national registries and exchange platforms. By contrast, OHAs seem more oriented to trade with a few counterparts, thus making the related system more fragmented.

**Table 2:** EU ETS network diagnostic. Columns labels refer to: number of nodes (#*N*); number of edges (#*E*); density (*dens*); reciprocity (*rec*); transitivity (*trans*); diameter (*d*); assortativity (*assort*). Centralization measures are indicated with symbol <*x*>, where *x* is the degree (*K*), the closeness (*C*), the betweenness (*B*) or the eigenvector centrality (*evcent*). Results refer to the period 2005-2012. Source: Authors’ own elaborations.

#N	#E	dens.	rec.	trans.	d	assort.	<*K*>	<*K*^*In*^>	<*K*^*Out*^>	<*C*>	<B>	<evcent>	subset
35	699	0.57	0.87	0.82	3	-0.13	0.38	0.35	0.38	0.16	0.08	0.34	All
34	680	0.59	0.86	0.82	3	-0.17	0.37	0.36	0.36	0.49	0.08	0.33	Trade
25	292	0.47	0.88	0.70	2	-0.25	0.47	0.47	0.43	0.21	0.08	0.44	All_PhaseI
35	692	0.56	0.87	0.82	3	-0.13	0.39	0.36	0.39	0.16	0.08	0.34	All_PhaseII
25	291	0.47	0.88	0.70	2	-0.27	0.47	0.47	0.43	0.21	0.08	0.44	Trade_PhaseI
34	673	0.58	0.86	0.82	3	-0.16	0.38	0.37	0.37	0.49	0.09	0.33	Trade_PhaseII
22	175	0.36	0.88	0.63	3	-0.25	0.45	0.48	0.48	0.47	0.16	0.49	Trade_PhaseI_PHA
27	427	0.59	0.89	0.72	2	-0.29	0.45	0.43	0.43	0.52	0.06	0.36	Trade_PhaseII_PHA
24	132	0.23	0.59	0.57	5	0.03	0.43	0.46	0.37	0.20	0.20	0.68	Trade_PhaseI_OHA
28	209	0.27	0.80	0.60	3	-0.05	0.50	0.46	0.50	0.09	0.19	0.60	Trade_PhaseII_OHA

Table 2 also shows that the EU ETS is a slightly disassortative network, meaning that counterparts usually tend to be connected with nodes dissimilar in terms of degree distribution, thus in line with other infrastructural networks (see e.g., [25–27]). This result is particularly evident in the PHAs specification, which is coherent with the activity carried out by this group: since this set of accounts mainly refers to financial intermediaries then diversification is more likely to occur and should actually be put in place by PHAs. Finally, centralization scores indicate the graph-level centrality for different centrality measures. Although the aforementioned centrality measures provide different perspectives of node centrality, our findings seem to depict the EU ETS as a more centralized network during Phase I. This reasonably reflects the presence of a few very central national registries during the first years of the program, while progressively the system became less polarized. For instance, Denmark and the Netherlands had favourable conditions to set up accounts during the early stages of the program, while other Member States such as France, Germany and the United Kingdom were among the few countries in Europe with dedicated exchange platforms for allowances. No less importantly, the centrality of some national registries may have been heavily influenced by carbon carousel frauds such as that occurred in the France’s Bluenext exchange in June 2009 [28, 29], which weakened the platform and contributed to its closure at the end of 2012. For instance, such episodes affected transferred volumes through France, placing this node as a very active player during the VAT fraud but then limiting its centrality once France changed its VAT rules in 2009 to respond against the fraud.

### What would have been the EU ETS configuration without UK?

The topological investigation we will propose in this subsection offers a clear picture: the UK was involved in a huge portion of transactions which -if not performed via UK- would have been reassigned to the remaining registries producing a substantial reshuffle within the EU ETS. We can only advance some hypotheses on how these transactions might have been reassigned. We introduce three scenarios as milestones to investigate how the EU ETS would have been without UK.

The first scenario is the one obtained by simply removing all the transactions in which UK is a counterpart; this is a limit case where we assume that exchanging allowances with UK is the main reason for that trade, so that dropping UK determines the deletion of that transaction and the impossibility to perform the same trade via a different registry. The second scenario reassigns the share of UK transactions proportionally to its neighborhood; in this scenario, we hypothesize that UK plays an intermediary role between some registries and that allowances passing through UK can be reasonably reassigned to registries in its neighborhood according to their weight in the market share of UK. The third scenario is a purely agnostic approach in which, to verify whether some properties of the network are confirmed, we randomly reassign the bundle of UK transactions to other registries without specific assumptions about the way these allowances are reallocated. Table 3 summarizes the respective estimates.

**Table 3:** EU ETS network diagnostic: Alternative scenarios. Columns labels refer to: number of nodes (#*N*); number of edges (#*E*); density (*dens*); reciprocity (*rec*); transitivity (*trans*); diameter (*d*); assortativity (*assort*). Centralization measures are indicated with symbol <*x*>, where *x* is the degree (*K*), the closeness (*C*), the betweenness (*B*) or the eigenvector centrality (*evcent*). The first panel exhibits the *No Reassignment* scenario, the second panel shows the *Proportional* scenario, while the last panel reports the *Random* scenario. Results refer to the period 2005-2012. Source: Authors’ own elaborations.

#N	#E	dens.	rec.	trans.	d	assort.	<*K*>	<*K*^*In*^>	<*K*^*Out*^>	<*C*>	<B>	<evcent>	subset
*No Reassignment*													
34	635	0.55	0.86	0.82	4	-0.10	0.35	0.37	0.31	0.15	0.04	0.36	All
33	617	0.57	0.86	0.81	3	-0.13	0.35	0.38	0.29	0.15	0.05	0.35	Trade
24	250	0.43	0.87	0.68	2	-0.24	0.50	0.50	0.46	0.23	0.13	0.47	All_PhaseI
34	628	0.54	0.86	0.82	4	-0.09	0.34	0.35	0.32	0.13	0.06	0.36	All_PhaseII
24	249	0.43	0.87	0.68	2	-0.26	0.50	0.51	0.46	0.23	0.13	0.47	Trade_PhaseI
33	610	0.56	0.85	0.81	3	-0.12	0.34	0.36	0.30	0.13	0.06	0.35	Trade_PhaseII
21	147	0.33	0.88	0.59	3	-0.27	0.47	0.50	0.50	0.49	0.19	0.52	Trade_PhaseI_PHA
26	374	0.55	0.88	0.70	2	-0.27	0.46	0.46	0.46	0.56	0.07	0.39	Trade_PhaseII_PHA
23	115	0.22	0.61	0.53	5	0.02	0.43	0.45	0.36	0.21	0.24	0.69	Trade_PhaseI_OHA
27	176	0.24	0.77	0.58	4	-0.03	0.52	0.48	0.52	0.10	0.23	0.64	Trade_PhaseII_OHA
*Proportional*													
34	996	0.86	0.91	1.00	2	0.44	0.08	0.08	0.08	0.03	0.03	0.09	All
33	969	0.89	0.88	1.00	2	-0.08	0.07	0.08	0.08	0.01	0.00	0.09	Trade
24	439	0.76	0.88	0.97	2	0.17	0.17	0.20	0.12	0.08	0.02	0.19	All_PhaseI
34	996	0.86	0.91	1.00	2	0.44	0.08	0.08	0.08	0.03	0.03	0.09	All_PhaseII
24	438	0.76	0.88	0.97	2	0.18	0.17	0.21	0.12	0.08	0.02	0.19	Trade_PhaseI
33	969	0.89	0.88	1.00	2	-0.08	0.07	0.08	0.08	0.01	0.00	0.09	Trade_PhaseII
21	220	0.50	0.85	0.85	3	-0.03	0.40	0.48	0.33	0.51	0.15	0.39	Trade_PhaseI_PHA
26	676	1.00	1.00	1.00	1	na	0.00	0.00	0.00	0.00	0.00	0.00	Trade_PhaseII_PHA
23	138	0.26	0.61	0.67	5	0.10	0.40	0.41	0.36	0.19	0.15	0.64	Trade_PhaseI_OHA
27	317	0.43	0.87	0.88	3	0.18	0.37	0.32	0.39	0.08	0.10	0.40	Trade_PhaseII_OHA
*Random*													
34	1122	0.97	0.97	1.00	1	na	0.02	0.03	0.00	0.00	0.00	0.03	All
33	1024	0.94	0.94	1.00	1	na	0.03	0.03	0.03	0.00	0.00	0.03	Trade
24	576	1.00	1.00	1.00	1	na	0.00	0.00	0.00	0.00	0.00	0.00	All_PhaseI
34	1122	0.97	0.97	1.00	1	na	0.02	0.03	0.00	0.00	0.00	0.03	All_PhaseII
24	552	0.96	0.96	1.00	1	na	0.02	0.04	0.00	0.00	0.00	0.04	Trade_PhaseI
33	1024	0.94	0.94	1.00	1	na	0.03	0.03	0.03	0.00	0.00	0.03	Trade_PhaseII
21	420	0.95	0.95	1.00	1	na	0.03	0.00	0.05	0.00	0.00	0.00	Trade_PhaseI_PHA
26	676	1.00	1.00	1.00	1	na	0.00	0.00	0.00	0.00	0.00	0.00	Trade_PhaseII_PHA
23	484	0.91	0.91	1.00	1	na	0.05	0.04	0.04	0.00	0.00	0.05	Trade_PhaseI_OHA
27	729	1.00	1.00	1.00	1	na	0.00	0.00	0.00	0.00	0.00	0.00	Trade_PhaseII_OHA

The first panel in Table 3 shows the scenario obtained by simply removing UK and all the links in which UK is at least one of the counterpart of the transaction. Even in this case we notice a few differences between the *All* and the *Trade* specifications, and we confirm the increasing connectivity from Phase I to Phase II. More generally, the network appears slightly less dense and connected under this scenario with respect to the actual EU ETS representation reported in Table 2. Similarly, the centralization measures for both the *All* and the *Trade* specifications are usually lower than those computed for the original case. Interestingly, the partition based on each Phase indicates that previous result is the combined effect of a rise in Phase I and a drop in Phase II, thus suggesting that the central role of UK seems to have been more effective during Phase II than Phase I when other national registries were very pivotal as well. Also, the subset of only PHAs shows that the removal of UK increases the centralization measures in both Phases, while the OHAs specification appears much more stable with no substantial changes in the reported measures with and without the UK (cfr. Table 2). It is well-known, in fact, the important role played by a club of other national registries (e.g., Denmark, France, Germany, and the Netherlands) as key market places for trading allowances thanks to the presence of devoted exchange platforms and favourable set-up conditions. By dropping a competitor as UK, their role is further enhanced and they emerge even more clearly as very pivotal nodes, especially if we focus on PHAs which are more likely to represent financial intermediaries very active across these stock exchanges.

The second panel in Table 3 exhibits the case corresponding to the *Proportional* scenario. We assume that links to UK are assigned to each target node in the out-neighborhood of UK proportionally to its weight among all flows departing from the UK (i.e., its weight in the UK allowance exports flow); similarly, links exiting from UK are assigned to each source node in the in-neighborhood of UK in proportion to its weight in the in-strength of the UK (i.e., its weight in the UK allowance import flows). The network arising in this scenario is highly connected and dense. This is due to the fact that UK is involved in a significant share of transactions where it plays a role as a hub/intermediary between national registries otherwise poorly connected. By creating links between the in- and the out-neighborhood of UK, we replace the hub node represented by UK with links connecting almost every node. This occurs because UK is basically connected to each Member State of the EU ETS, which highlights the central role of UK in the program and explains why we get this very dense configuration under the *Proportional* scenario. Furthermore, we still observe the same regularities already commented about the increasing connectivity during Phase II with respect to Phase I. Note also that in this scenario the assortativity coefficient is often positive, meaning that transferring and acquiring counterparts are here much more similar than in the original case (i.e., when connected via UK). Remarkably, when we circumscribe the analysis to only PHAs, the system becomes totally connected in Phase II, thus emphasizing the role of UK as a key player in facilitating trades among market participants spread in the EU ETS. Finally, we remark that the system without UK and with proportional reassignment is very uniform as indicated by the centralization measures.

We also propose a basic *Random* scenario in which UK’s links are randomly reassigned to the remaining pairs of registries. Results in the third panel of Table 3 indicate a well-connected system in line with the discussion for the *Proportional* scenario. Hence, if those transactions originally performed via UK would be reassigned to the remaining nodes either proportionally to their weight in the UK’s neighborhood or even randomly, still we will get a more uniform and connected network than the actual EU ETS. A peculiar result emerges in the *Random* scenario if we focus on only OHAs: randomization allows to bypass some kind of country-barriers that force transactions for liable installations to be biased towards domestic transactions or a few other registries. Finally, as expected due to the relevant amount of transactions involving UK, their random reassignment is able to basically generate a network configuration that is weakly structured. The removal of UK could be interpreted as a shock to the system: indeed, the agnostic reassignment of the UK-related transactions without any particular rule is likely to generate a significant perturbation which seems able to modify substantially the original configuration of the network.

### Winners and losers from the removal of UK

Once a very central node like UK is dropped from the system, links will be reorganized, the centrality of the remaining nodes might result reshuffled, and the overall structure of the system may eventually change. The topological investigation discussed in the previous subsection suggests that in each of the three alternative scenarios, the removal of UK’s transactions significantly affects the configuration of the network. This subsection discusses the topological impact at the level of single nodes to detect which registries would be, eventually, more affected by such reassignment. Some registries could gain positions in the centrality rankings becoming more influential in the network, while others may reach even more peripheral positions once UK is removed. The former can be seen as the “winners” who gain from removing the UK node, while the latter are the “loosers” who, conversely, achieve even more marginal roles in the system.

To perform such analysis, the first panel of Table 4 focuses on observations related only to Phase II to provide a representation of the most recently concluded EU ETS phase (Phase III being still on-going). It also refers to the pure *Trade* specification because the other types of transactions, such as the issuance, allocation and surrendering of allowances, are more country-specific and affected by the relationships with governmental counterparts. Instead, the second panel of Table 4 refers to those transactions involving only PHAs to further verify variations in centrality scores among those accounts (mainly financial intermediaries, banks, and brokers) for which is easier to switch across different national registries.

**Table 4:** Network centrality statistics. This table reports the following scenarios: the actual *EU ETS* (I), *No Reassignment* (II), *Proportional* (III), and *Random* (IV). Data refer to Phase II. The first panel includes both internal and external transactions (*Trade* specification). The second panel refers to PHAs only. Notice that due to the presence of some registries poorly connected with the rest of the system, centrality measures for some nodes appear higher than those for the others. Source: Authors’ own elaborations.

*Nodes*	*degree*				*in* − *degree*				*out* − *degree*				*strength*				*in* − *strength*			
	I	II	III	IV	I	II	III	IV	I	II	III	IV	I	II	III	IV	I	II	III	IV
*United Kingdom*	64				32				32				0.284				0.291			
Austria	49	47	63	64	25	24	31	32	24	23	32	32	0.005	0.007	0.007	0.012	0.006	0.008	0.008	0.013
Belgium	50	48	62	64	27	26	31	32	23	22	31	32	0.013	0.018	0.017	0.021	0.013	0.019	0.017	0.021
Bulgaria	40	38	62	64	18	17	31	32	22	21	31	32	0.003	0.005	0.004	0.010	0.003	0.004	0.004	0.009
Cyprus	6	4	62	64	3	2	31	32	3	2	31	32	0.000	0.000	0.000	0.006	0.000	0.000	0.000	0.006
Czech Republic	49	47	63	64	22	21	31	32	27	26	32	32	0.017	0.025	0.023	0.026	0.016	0.023	0.022	0.025
Denmark	60	58	62	64	31	30	31	32	29	28	31	32	0.063	0.090	0.086	0.079	0.063	0.090	0.088	0.079
Estonia	43	41	62	64	20	19	31	32	23	22	31	32	0.001	0.002	0.002	0.008	0.001	0.001	0.001	0.007
European Commission	17	16	38	64	7	7	7	32	10	9	31	32	0.000	0.001	0.001	0.007	0.000	0.001	0.000	0.007
Finland	44	42	63	64	25	24	31	32	19	18	32	32	0.009	0.013	0.013	0.017	0.010	0.013	0.015	0.017
France	60	58	62	64	31	30	31	32	29	28	31	32	0.177	0.250	0.246	0.207	0.179	0.253	0.252	0.210
Germany	59	57	62	64	30	29	31	32	29	28	31	32	0.170	0.236	0.239	0.196	0.173	0.241	0.248	0.200
Greece	29	27	62	64	15	14	31	32	14	13	31	32	0.002	0.002	0.003	0.008	0.002	0.002	0.002	0.008
Hungary	37	35	62	64	17	16	31	32	20	19	31	32	0.006	0.008	0.008	0.013	0.005	0.008	0.007	0.013
Iceland	2	1	31	32	2	1	31	32	0	0	0	0	0.000	0.000	0.000	0.003	0.000	0.000	0.000	0.006
Ireland	34	32	62	64	17	16	31	32	17	16	31	32	0.008	0.012	0.012	0.016	0.008	0.012	0.012	0.015
Italy	56	54	62	64	28	27	31	32	28	27	31	32	0.027	0.037	0.039	0.036	0.028	0.039	0.040	0.037
Latvia	31	29	62	64	14	13	31	32	17	16	31	32	0.001	0.001	0.002	0.007	0.001	0.001	0.002	0.007
Liechtenstein	45	43	62	64	22	21	31	32	23	22	31	32	0.003	0.004	0.004	0.010	0.003	0.004	0.004	0.009
Lithuania	30	28	62	64	12	11	31	32	18	17	31	32	0.001	0.001	0.002	0.007	0.001	0.001	0.001	0.007
Luxembourg	27	25	63	64	15	14	31	32	12	11	32	32	0.001	0.001	0.001	0.007	0.001	0.001	0.001	0.007
Malta	3	2	33	64	2	1	32	32	1	1	1	32	0.000	0.000	0.000	0.006	0.000	0.000	0.000	0.006
Netherlands	60	58	62	64	31	30	31	32	29	28	31	32	0.080	0.114	0.109	0.098	0.081	0.116	0.113	0.100
Norway	41	39	62	64	23	22	31	32	18	17	31	32	0.004	0.005	0.006	0.010	0.004	0.005	0.006	0.010
RoW	36	34	62	64	18	17	31	32	18	17	31	32	0.011	0.010	0.018	0.014	0.005	0.005	0.007	0.010
Poland	53	51	62	64	27	26	31	32	26	25	31	32	0.013	0.019	0.017	0.021	0.012	0.017	0.016	0.020
Portugal	32	30	63	64	18	17	31	32	14	13	32	32	0.008	0.012	0.010	0.016	0.008	0.012	0.010	0.016
Romania	43	41	62	64	20	19	31	32	23	22	31	32	0.022	0.033	0.029	0.033	0.021	0.032	0.027	0.032
Slovakia	48	46	63	64	24	23	31	32	24	23	32	32	0.003	0.005	0.005	0.010	0.003	0.004	0.004	0.009
Slovenia	37	35	62	64	19	18	31	32	18	17	31	32	0.001	0.002	0.001	0.007	0.001	0.002	0.001	0.007
Spain	50	48	63	64	26	25	31	32	24	23	32	32	0.045	0.067	0.059	0.060	0.047	0.070	0.061	0.063
Sweden	47	45	62	64	26	25	31	32	21	20	31	32	0.003	0.004	0.004	0.009	0.003	0.004	0.004	0.010
Switzerland	54	52	62	64	26	25	31	32	28	27	31	32	0.016	0.015	0.029	0.018	0.013	0.012	0.024	0.015
Ukraine	10	9	31	32	0	0	0	0	10	9	31	32	0.002	0.001	0.005	0.004	0.000	0.000	0.000	0.000
**Only PHAs** ↓																				
*United Kingdom*	54				27				27				0.128				0.128			
Austria	32	30	52	52	17	16	26	26	15	14	26	26	0.004	0.005	0.005	0.013	0.004	0.005	0.005	0.013
Belgium	22	20	52	52	11	10	26	26	11	10	26	26	0.004	0.004	0.005	0.012	0.004	0.004	0.004	0.012
Bulgaria	21	19	52	52	10	9	26	26	11	10	26	26	0.000	0.000	0.000	0.010	0.000	0.000	0.000	0.009
Czech Republic	35	33	52	52	16	15	26	26	19	18	26	26	0.013	0.015	0.015	0.020	0.012	0.014	0.014	0.020
Denmark	53	51	52	52	27	26	26	26	26	25	26	26	0.154	0.187	0.169	0.150	0.154	0.186	0.170	0.150
Estonia	32	30	52	52	15	14	26	26	17	16	26	26	0.001	0.002	0.001	0.011	0.001	0.002	0.001	0.011
Finland	23	21	52	52	13	12	26	26	10	9	26	26	0.003	0.002	0.004	0.011	0.003	0.002	0.005	0.011
France	49	47	52	52	24	23	26	26	25	24	26	26	0.408	0.493	0.449	0.382	0.403	0.486	0.444	0.376
Germany	52	50	52	52	26	25	26	26	26	25	26	26	0.169	0.183	0.202	0.147	0.176	0.190	0.211	0.153
Greece	23	21	52	52	11	10	26	26	12	11	26	26	0.001	0.000	0.001	0.010	0.000	0.000	0.001	0.010
Hungary	18	16	52	52	9	8	26	26	9	8	26	26	0.001	0.001	0.001	0.010	0.001	0.001	0.001	0.010
Ireland	17	15	52	52	8	7	26	26	9	8	26	26	0.002	0.001	0.003	0.010	0.002	0.001	0.004	0.010
Italy	39	37	52	52	20	19	26	26	19	18	26	26	0.018	0.014	0.025	0.020	0.017	0.016	0.022	0.021
Latvia	16	14	52	52	8	7	26	26	8	7	26	26	0.000	0.000	0.000	0.010	0.000	0.000	0.000	0.009
Liechtenstein	40	38	52	52	19	18	26	26	21	20	26	26	0.006	0.007	0.006	0.015	0.006	0.007	0.007	0.014
Lithuania	16	14	52	52	6	5	26	26	10	9	26	26	0.000	0.000	0.000	0.009	0.000	0.000	0.000	0.009
Luxembourg	17	15	52	52	8	7	26	26	9	8	26	26	0.000	0.000	0.000	0.010	0.000	0.000	0.000	0.010
Netherlands	53	51	52	52	26	25	26	26	27	26	26	26	0.047	0.042	0.062	0.041	0.048	0.042	0.064	0.041
Norway	27	25	52	52	14	13	26	26	13	12	26	26	0.002	0.001	0.003	0.010	0.002	0.001	0.002	0.010
Poland	41	39	52	52	21	20	26	26	20	19	26	26	0.009	0.011	0.010	0.018	0.009	0.011	0.010	0.018
Portugal	22	20	52	52	13	12	26	26	9	8	26	26	0.002	0.002	0.003	0.011	0.002	0.002	0.003	0.011
Romania	32	30	52	52	16	15	26	26	16	15	26	26	0.002	0.002	0.003	0.011	0.002	0.002	0.002	0.011
Slovakia	31	29	52	52	17	16	26	26	14	13	26	26	0.002	0.003	0.002	0.011	0.002	0.003	0.002	0.011
Slovenia	19	17	52	52	10	9	26	26	9	8	26	26	0.000	0.000	0.001	0.010	0.000	0.000	0.000	0.010
Spain	33	31	52	52	17	16	26	26	16	15	26	26	0.021	0.024	0.025	0.027	0.021	0.024	0.025	0.028
Sweden	37	35	52	52	18	17	26	26	19	18	26	26	0.002	0.002	0.003	0.011	0.002	0.002	0.002	0.011
*Nodes*	*out* − *degree*				*closeness*				*betweenness*				*eigenvector* *centrality*							
	I	II	III	IV	I	II	III	IV	I	II	III	IV	I	II	III	IV				
*United Kingdom*	0.276				1.000				1.000				1.000							
Austria	0.004	0.006	0.006	0.011	0.805	0.451	1.000	1.000	0.098	0.147	1.000	na	0.892	0.893	0.966	1.000				
Belgium	0.013	0.018	0.017	0.020	0.846	0.464	1.000	1.000	0.108	0.180	0.000	na	0.894	0.895	0.966	1.000				
Bulgaria	0.003	0.005	0.005	0.010	0.733	0.427	1.000	1.000	0.025	0.041	0.000	na	0.647	0.635	0.966	1.000				
Cyprus	0.000	0.000	0.000	0.006	0.516	0.327	1.000	1.000	0.000	0.000	0.000	na	0.088	0.045	0.966	1.000				
Czech Republic	0.018	0.026	0.024	0.027	0.846	0.464	1.000	1.000	0.091	0.138	1.000	na	0.776	0.770	0.966	1.000				
Denmark	0.063	0.090	0.084	0.079	0.917	0.485	1.000	1.000	0.217	0.358	0.000	na	0.996	1.000	0.966	1.000				
Estonia	0.002	0.003	0.003	0.009	0.786	0.444	1.000	1.000	0.048	0.082	0.000	na	0.698	0.689	0.966	1.000				
European Commission	0.000	0.001	0.001	0.007	0.673	0.400	0.970	1.000	0.008	0.014	0.000	na	0.235	0.247	0.231	1.000				
Finland	0.009	0.013	0.012	0.016	0.805	0.451	1.000	1.000	0.084	0.130	1.000	na	0.872	0.871	0.966	1.000				
France	0.176	0.247	0.240	0.205	0.917	0.485	1.000	1.000	0.217	0.358	0.000	na	0.996	1.000	0.966	1.000				
Germany	0.167	0.231	0.229	0.193	0.892	0.478	1.000	1.000	0.193	0.317	0.000	na	0.986	0.989	0.966	1.000				
Greece	0.002	0.003	0.004	0.008	0.660	0.400	1.000	1.000	0.005	0.008	0.000	na	0.559	0.544	0.966	1.000				
Hungary	0.006	0.009	0.008	0.013	0.717	0.421	1.000	1.000	0.020	0.034	0.000	na	0.620	0.608	0.966	1.000				
Iceland	0.000	0.000	0.000	0.000	0.516	0.327	0.970	1.000	0.000	0.000	0.000	na	0.083	0.041	0.966	1.000				
Ireland	0.008	0.012	0.012	0.016	0.673	0.405	1.000	1.000	0.021	0.035	0.000	na	0.614	0.602	0.966	1.000				
Italy	0.027	0.035	0.039	0.034	0.868	0.471	1.000	1.000	0.369	1.000	0.000	na	0.936	0.937	0.966	1.000				
Latvia	0.001	0.002	0.002	0.008	0.673	0.405	1.000	1.000	0.014	0.023	0.000	na	0.537	0.520	0.966	1.000				
Liechtenstein	0.003	0.005	0.004	0.010	0.805	0.451	1.000	1.000	0.076	0.125	0.000	na	0.762	0.754	0.966	1.000				
Lithuania	0.002	0.002	0.003	0.007	0.688	0.405	1.000	1.000	0.017	0.026	0.000	na	0.435	0.412	0.966	1.000				
Luxembourg	0.000	0.001	0.001	0.007	0.673	0.400	1.000	1.000	0.028	0.040	1.000	na	0.505	0.485	0.966	1.000				
Malta	0.000	0.000	0.000	0.006	0.508	0.030	0.970	1.000	0.000	0.000	0.000	na	0.046	0.000	1.000	1.000				
Netherlands	0.078	0.112	0.105	0.096	0.917	0.485	1.000	1.000	0.217	0.358	0.000	na	0.996	1.000	0.966	1.000				
Norway	0.003	0.004	0.005	0.009	0.767	0.438	1.000	1.000	0.037	0.062	0.000	na	0.817	0.813	0.966	1.000				
RoW	0.017	0.014	0.029	0.018	0.805	0.444	1.000	1.000	0.031	0.050	0.000	na	0.641	0.628	0.966	1.000				
Poland	0.014	0.020	0.018	0.023	0.868	0.471	1.000	1.000	0.123	0.201	0.000	na	0.879	0.878	0.966	1.000				
Portugal	0.008	0.012	0.011	0.016	0.717	0.421	1.000	1.000	0.037	0.055	1.000	na	0.649	0.637	0.966	1.000				
Romania	0.023	0.035	0.030	0.034	0.767	0.438	1.000	1.000	0.038	0.060	0.000	na	0.733	0.726	0.966	1.000				
Slovakia	0.004	0.006	0.005	0.011	0.805	0.451	1.000	1.000	0.102	0.154	1.000	na	0.839	0.836	0.966	1.000				
Slovenia	0.001	0.001	0.001	0.007	0.717	0.421	1.000	1.000	0.027	0.045	0.000	na	0.687	0.677	0.966	1.000				
Spain	0.043	0.064	0.057	0.057	0.846	0.464	1.000	1.000	0.152	0.238	1.000	na	0.866	0.864	0.966	1.000				
Sweden	0.003	0.004	0.004	0.009	0.825	0.457	1.000	1.000	0.070	0.116	0.000	na	0.890	0.890	0.966	1.000				
Switzerland	0.020	0.018	0.033	0.021	0.868	0.471	1.000	1.000	0.262	0.644	0.000	na	0.895	0.893	0.966	1.000				
Ukraine	0.005	0.003	0.009	0.008	0.589	0.364	0.970	1.000	0.000	0.000	0.000	na	0.000	0.000	0.000	0.000				
**Only PHAs** ↓																				
*United Kingdom*	0.129				1.000				1.000				1.000							
Austria	0.004	0.005	0.006	0.013	0.722	0.714	1.000	1.000	0.089	0.089	na	na	0.730	0.723	1.000	1.000				
Belgium	0.004	0.004	0.005	0.012	0.650	0.641	1.000	1.000	0.036	0.037	na	na	0.477	0.450	1.000	1.000				
Bulgaria	0.000	0.000	0.000	0.010	0.650	0.641	1.000	1.000	0.011	0.011	na	na	0.458	0.432	1.000	1.000				
Czech Republic	0.014	0.015	0.016	0.021	0.765	0.758	1.000	1.000	0.137	0.138	na	na	0.702	0.693	1.000	1.000				
Denmark	0.154	0.188	0.168	0.151	1.000	1.000	1.000	1.000	0.941	1.000	na	na	1.000	1.000	1.000	1.000				
Estonia	0.001	0.002	0.001	0.011	0.743	0.735	1.000	1.000	0.114	0.116	na	na	0.646	0.631	1.000	1.000				
Finland	0.003	0.002	0.004	0.011	0.667	0.658	1.000	1.000	0.042	0.043	na	na	0.529	0.503	1.000	1.000				
France	0.413	0.500	0.453	0.387	0.963	0.962	1.000	1.000	0.628	0.647	na	na	0.935	0.935	1.000	1.000				
Germany	0.161	0.175	0.193	0.142	0.963	0.962	1.000	1.000	0.828	0.863	na	na	0.980	0.980	1.000	1.000				
Greece	0.001	0.001	0.002	0.010	0.667	0.658	1.000	1.000	0.032	0.032	na	na	0.500	0.477	1.000	1.000				
Hungary	0.001	0.001	0.002	0.010	0.619	0.610	1.000	1.000	0.009	0.009	na	na	0.430	0.403	1.000	1.000				
Ireland	0.002	0.001	0.003	0.010	0.619	0.610	1.000	1.000	0.000	0.000	na	na	0.398	0.370	1.000	1.000				
Italy	0.018	0.012	0.028	0.018	0.812	0.806	1.000	1.000	0.249	0.254	na	na	0.829	0.827	1.000	1.000				
Latvia	0.000	0.000	0.000	0.010	0.605	0.595	1.000	1.000	0.004	0.004	na	na	0.362	0.328	1.000	1.000				
Liechtenstein	0.006	0.007	0.006	0.015	0.897	0.893	1.000	1.000	0.330	0.339	na	na	0.754	0.742	1.000	1.000				
Lithuania	0.000	0.000	0.000	0.010	0.605	0.595	1.000	1.000	0.009	0.009	na	na	0.265	0.222	1.000	1.000				
Luxembourg	0.000	0.000	0.000	0.010	0.619	0.610	1.000	1.000	0.007	0.007	na	na	0.386	0.355	1.000	1.000				
Netherlands	0.045	0.041	0.060	0.041	1.000	1.000	1.000	1.000	0.908	0.952	na	na	0.980	0.980	1.000	1.000				
Norway	0.002	0.001	0.004	0.010	0.703	0.694	1.000	1.000	0.061	0.061	na	na	0.594	0.575	1.000	1.000				
Poland	0.009	0.011	0.010	0.017	0.812	0.806	1.000	1.000	0.355	0.365	na	na	0.839	0.834	1.000	1.000				
Portugal	0.002	0.002	0.002	0.011	0.667	0.658	1.000	1.000	0.007	0.006	na	na	0.591	0.575	1.000	1.000				
Romania	0.003	0.002	0.003	0.011	0.765	0.758	1.000	1.000	0.129	0.128	na	na	0.692	0.682	1.000	1.000				
Slovakia	0.002	0.003	0.002	0.012	0.722	0.714	1.000	1.000	0.062	0.061	na	na	0.718	0.709	1.000	1.000				
Slovenia	0.001	0.000	0.001	0.010	0.634	0.625	1.000	1.000	0.030	0.032	na	na	0.463	0.437	1.000	1.000				
Spain	0.021	0.023	0.026	0.027	0.743	0.735	1.000	1.000	0.150	0.150	na	na	0.720	0.711	1.000	1.000				
Sweden	0.003	0.002	0.004	0.011	0.765	0.758	1.000	1.000	0.228	0.229	na	na	0.740	0.731	1.000	1.000				

As shown in Table 4, we note that the UK is a very central node, while the club of the other key nodes usually encompasses: Denmark, France, Germany, the Netherlands and sometimes Italy. Together with the UK, these registries form a core of very connected nodes surrounded by a cloud of registries related to peripheral countries within the EU ETS. Among the latter it is clear that the UK plays for them a role as hub/intermediary between these nodes otherwise poorly connected, so the removal of UK without the reassignment of its links is likely to reduce the connectivity of these registries with the rest of the system. Conversely, those already very central nodes usually appear even more central once the UK and its links are removed.

The first three blocks in Table 4 refer to degree and its variants (in-degree and out-degree). These indicators provide a simple representation of the network configuration based on a binary view which assigns links regardless the transferred amount. This basic perspective is helpful for two reasons: i) it clearly indicates that Denmark, France, Germany, Italy, the Netherlands and the United Kingdom are key counterparts in the system being connected to almost every registry; ii) conversely, there is a cloud of less central registries mostly related to geographically peripheral countries. Only a few differences appear between the first and second panel of the table; however, when we circumscribe the analysis to only PHAs (bottom panel), fewer active registries are present and some of them, e.g. Austria, Italy or Spain, appear less active compared to the configuration including the other account types (top panel).

A more effective representation of the EU ETS is offered by the second block of the topological measures (namely, strength, in-strength and out-strength). In the actual EU ETS configuration (case I), the UK is involved in a significant portion of transactions, although other registries are also very active either in terms of transferring or acquiring operations. France and Germany, for instance, would be the most central nodes in the network once the UK is removed, while those registries in the periphery would continue to play a marginal role. In the PHAs specification, the UK is not the most central node and the reassignment of its links clearly identifies France as the key node in the network under all the alternative scenarios. More specifically, the *Random* scenario (case IV) penalizes very central nodes (e.g., Denmark, France, and Germany) with respect to the actual EU ETS configuration, while the *Proportional* scenario (case III) coincides with a gain in centrality for these registries. The latter are relevant transferring and acquiring counterparts for the UK and would proportionally receive the lion’s share of its transactions once the UK is removed.

Subsequent blocks of Table 4 present centrality indicators more related to the overall network and the way each node is connected to the rest of the system. These measures may not be necessarily positively correlated between each other [30]. An example about the relationships between these centrality measures under each alternative scenario is presented in S1 Fig. Closeness can be interpreted as a measure of how long it will take to spread information from a certain node to all the other nodes sequentially. In the first panel, the UK is among the most central nodes in terms of closeness. Some geographically peripheral registries (e.g., Cyprus, Malta, Iceland, and Ukraine) are more distant from the rest of the system, while in general only a few links are needed to connect each node to the others. Almost all registries are connected to the others on average by a couple of steps. Instead, as expected, values for closeness measures would fall if we remove the UK and we do not reassign the corresponding links (case II), while they would increase if we reassign them proportionally to its neighborhood (case III). Overall, this finding confirms that the UK facilitates connections among different parts of the EU ETS. Configurations for only PHAs are dense and highly connected with the UK playing a prominent role, although other registries are very central and remain so even if we drop the UK without reassigning its links. Hence, within the PHAs, the system appears well connected and removing the UK does not significantly reduce the distance between registries.

Betweenness indicates how frequently a node lies along the geodesic pathways connecting other nodes, thus representing an asymmetric measure of centrality. The UK is the most central node in this framework, thus emphasizing its role as hub/intermediary between different parts of the network. Denmark, France, Germany, Italy, the Netherlands and Switzerland form a club of central nodes and they benefit more than others from the drop of the UK. Their centrality scores, although higher than those of most other registries, are far from the UK’s value, thus supporting the interpretation that the latter is the only key node in that framework. Instead, if we focus on PHAs only, other nodes appear very central: Denmark, Germany, and the Netherlands are, in fact, almost as central as the UK, while most of the remaining nodes are peripheral.

Finally, we consider the eigenvector centrality. Again the club composed by Denmark, France, Germany, Italy, the Netherlands and UK reach very high central scores, while in the bottom part of the ranking there are those geographically peripheral countries already seen in the previous centrality measures. The eigenvector is an appealing indicator of centrality since it does not only consider the amount of flows impacting to a certain node (as already measured, e.g., by the strength), but it also consider the structure of the network and, in particular, of the nearest nodes from and to which the node operates transactions. Hence, it is worth remarking that central nodes in terms of eigenvector are not necessarily related to registries with high inflows (see, e.g., the high values of the eigenvector centrality for Austria, Finland or Slovakia). In general, removing the UK without reassigning its link causes peripheral nodes to become slightly more marginal, while for more central nodes the effect is spurious. In the PHAs specification, the ranking is instead more clear, especially in the upper tail of the distribution. Denmark, France, Germany, and the Netherlands are the most central nodes together with the UK, and the removal of the latter node (without reassignment) basically decreases only the centrality scores of the remaining less central nodes. *Proportional* and *Random* scenarios are almost fully connected networks, thus the indicator reaches its maximum value.

The second panel of Table 4 is likely to represent the most plausible scenario arising from the removal of the UK, since it deals with non-liable entities (namely, PHAs) that can easily switch into a different national registry for trading purposes. This subsection suggests that removing the UK may induce non-liable entities to move from the UK to already very central registries, which are also characterized by the presence of devoted exchanges for trading allowances.

## Discussion

The UK has always played a pivotal role in the EU ETS: it is the second-largest GHG emitter in the EU and has long been one of the most ambitious countries in terms of climate policies and targets within the EU. The UK ETS was the first, multi-sector emission trading program and its experience somehow inspired the EU ETS. For all these reasons, if the UK decides to leave the EU ETS after Brexit, this will obviously have significant impacts on the EU ETS (though these might as well be smaller than those on the UK itself).

This study exploits network analysis tools to assess the role played by the UK in the EU ETS and to compare the actual structure of the system (including the UK) with the one that would have emerged without the UK under different scenarios. In particular, in the (basic but probably most realistic) proportionality scenario we evaluate how the structure would change if the large import and export flows involving the UK registry were reassigned to its partners in proportion to their weight in the UK relationships.

When the UK is removed from the system the structure of the network turns out to change deeply. Indeed, in some of the configurations taken into account (e.g. the *Trade* specification that encompasses both internal and external transactions) the UK was basically an outlier. In these cases the departure of the UK would transform the network from an almost star-like system (the UK being at the centre of the star and its partners surrounding it) to a core-periphery structure with a club of core countries (Denmark, France, Germany, Netherlands, partly Italy) becoming more central in the network while the others remain at the periphery of the system. As one would expect, therefore, the structure of the EU ETS is not persistent to a large shock such as the UK exit from the system. However, this does not seem to apply to the network composed of PHAs only. In fact, the PHAs network is already very connected and more homogeneous and it is likely to remain so, with or without the UK. This reflects the very nature of PHAs which, being mainly financial intermediaries, are more likely to trade across national borders, thus establishing links across all nodes within the PHAs network.

## Supporting information

S1 Fig In- vs. out- strength distributions. Plot shows the distributions of in-strength vs. out-strength in Phase II. Panel *a*) is the *All* case; *b*) is the *Trade* case; *c*) is the *Trade* case for only OHAs; *d*) is the *Trade* case for only PHAs. Colors refer to: the actual EU ETS (designated with purple); the *No reassignment* case (in red); the *Proportional case* (in green); and the *Random* case (in blue). Only very central nodes are highlighted in color, while the orthogonal dotted lines refer to UK under the actual EU ETS network and are introduced as a reference point. Source: Authors’ own elaborations.
(PDF)
