# Supplementary material for: With or without U(K): A pre-Brexit network analysis of the EU ETS
Source: PLoS One. 2019 Sep 9;14(9):e0221587. doi: 10.1371/journal.pone.0221587 (PMC6733441; doi:10.1371/journal.pone.0221587)

Supporting information: Winners vs. Losers: With or without U(K): a pre-Brexit network analysis of the EU ETS

Simone Borghesi[1,2]*, Andrea Flori[3]

1 FSR Climate, European University Institute, Florence, Italy
2 Department of Political and International Sciences, University of Siena, Siena, Italy
3 Department of Management, Economics and Industrial Engineering, Polytechnic University of Milan, Milan, Italy

* simone.borghesi@eui.eu

S1. In- vs. Out- Strength comparison.

Fig S1 exhibits some examples about the relationships between centrality measures under different scenarios. To simply represent the centrality direction of the transfers we plot, in particular, the in-strength vs. the out-strength of each registry node. The relationship is almost linear and many registries basically cancel out their inflows with a similar amount of outflows. The first plot on the top-left position (plot *a*) considers all types of transactions during Phase II: the two nodes that gain more from the removal of the UK are France and Germany. This result is also supported by the *Trade* case (plot *b* in the top-right of the figure) where France, Germany together with Denmark and the Netherlands reach more central positions in the network once the UK is removed. Finally, plots in the bottom-panel show these relationships for only OHAs (plot *c*) or only PHAs (plot *d*). In the OHAs case, the UK is not so central compared to Germany or Spain which become even more central in the network after the removal of UK. In the PHAs case we observe that those registries usually associated to the presence of exchange platforms for trading allowances become very central after the removal of the UK and the proportional reassignment of its links.

S1 Fig. In- vs. out- strength Distributions. Plot shows the distributions of in-strength vs. out-strength in Phase II. Panel *a*) is the *All* case; *b*) is the *Trade* case; *c*) is the *Trade* case for only OHAs; *d*) is the *Trade* case for only PHAs. Colors refer to: the actual EU ETS (designated with purple); the *No reassignment* case (in red); the *Proportional case* (in green); and the *Random* case (in blue). Only very central nodes are highlighted in color, while the orthogonal dotted lines refer to UK under the actual EU ETS network and are introduced as a reference point. Source: Authors' own elaborations.

(a) *All* case - Phase II

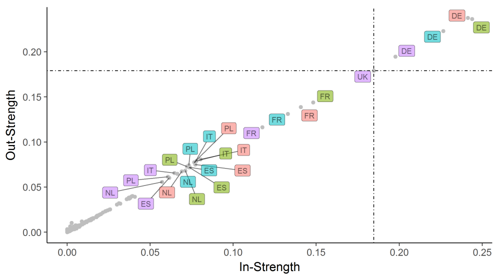

(b) *Trade* case - Phase II

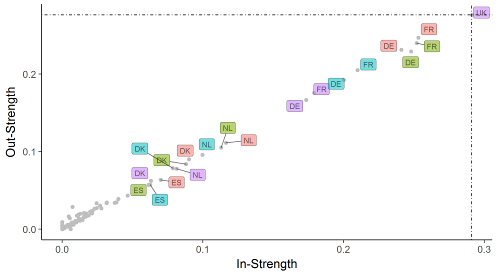

(c) *Trade* case - Phase II, only OHAs

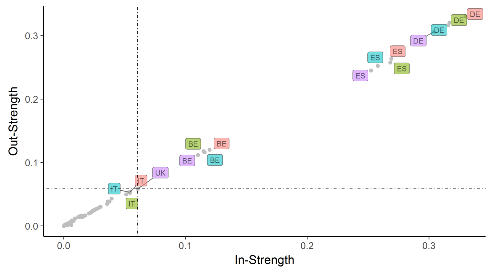

(d) *Trade* case - Phase II, only PHAs

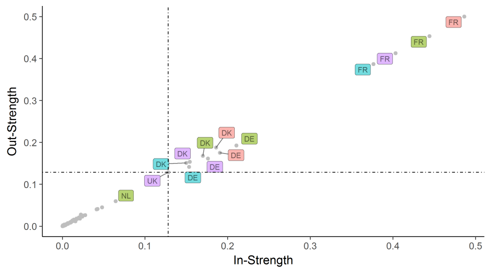

Supplement: S1 Fig — Plot shows the distributions of in-strength vs. out-strength in Phase II. Panel a) is the All case; b) is the Trade case; c) is the Trade case for only OHAs; d) is the Trade case for only PHAs. Colors refer to: the actual EU ETS (designated with purple); the No reassignment case (in red); the Proportional case (in green); and the Random case (in blue). Only very central nodes are highlighted in color, while the orthogonal dotted lines refer to UK under the actual EU ETS network and are introduced as a reference point. Source: Authors’ own elaborations. (PDF) [file pone.0221587.s001.pdf]
